# *Lactobacillus rhamnosus* GR-1 Attenuates Induction of Hypertrophy in Cardiomyocytes but Not through Secreted Protein MSP-1 (p75)

**DOI:** 10.1371/journal.pone.0168622

**Published:** 2017-01-13

**Authors:** Grace Ettinger, Jeremy P. Burton, Gregory B. Gloor, Gregor Reid

**Affiliations:** 1 Lawson Health Research Institute, London, Ontario, Canada; 2 Department of Microbiology and Immunology, University of Western Ontario, London, Canada; 3 Department of Surgery (Urology), University of Western Ontario, London, Canada; 4 Department of Biochemistry, University of Western Ontario, London, Canada; University of Cincinnati College of Medicine, UNITED STATES

## Abstract

Previous animal studies have shown that the administration of probiotic *Lactobacillus rhamnosus* can provide a protective effect against ischemia/reperfusion and necrotic injury to the intestine, liver, and heart, as well as a therapeutic effect to the outcome of ischemic injury to the heart, including cardiac hypertrophy and heart failure. We hypothesized that *L*. *rhamnosus* GR-1 major secreted protein 1 (MSP-1), also known as p75, plays a major role in this phenomenon. Experiments using neonatal rat ventricular cardiomyocytes showed that live and dead GR-1 bacteria, probiotic-conditioned media, and other probiotic species and strains inhibited the α1-adrenergic receptor agonist phenylephrine-induced hypertrophy as assessed by markers atrial natriuretic peptide and α-skeletal actin. However, using a mutant strain, we showed that this MSP-1 was not required for the inhibition. The ability of factors produced by lactobacilli to improve cardiac function warrants further study for the management of cardiac hypertrophy and heart failure.

## Introduction

In a previous study, we reported that *L*. *rhamnosus* GR-1 significantly improved the outcome after ischemia-induced heart failure in rats, by administering the probiotics in the drinking water daily for 4–6 weeks [1]. One of the main parameters of heart failure measured in this study was cardiac hypertrophy, which was significantly attenuated by the administration of probiotics [1]. This raised the question of what might be inducing the effect. Studies involving secreted proteins from *Lactobacillus rhamnosus* have shown to prevent cytokine-induced apoptosis in intestinal epithelial cells through Akt pathway activation and p38 MAPK inhibition [2]. Soluble factors recovered from *L*. *rhamnosus* GG (LGG) supernatant also exhibited this ability to prevent cytokine-mediated cell apoptosis, as well as induce cytoprotection through HSP induction [2]. Efforts to purify and characterize the soluble factors in LGG supernatant elucidated 2 proteins that promote cell growth and prevent apoptosis: p75 and p40 (named after their approximate weight in kD) [3,4]. Functional analyses characterized the p75 protein as a glycoside hydrolase [4]. Pre-treatment of p75 purified from LGG was reported to alleviate the I/R-induced heart tissue infarction in rats, by inhibiting I/R-induced HSP70 suppression [5]. In addition, administration of the probiotic drink Goodbelly (containing *L*. *plantarum* 299v) significantly reduced the severity of myocardial infarction following I/R surgery in rats [6].

Taken together, these data suggested an interesting potential for soluble factors produced by *Lactobacillus spp*. to protect against ischemic injury and ischemia-induced hypertrophy in cardiomyocytes. It is important to note, in the hepatic I/R and both the heart I/R studies, rats were administered the probiotic supplements by oral gavage, as a bolus, or in their drinking water. This indicates that despite the physical barrier of the intestine, the protective factor is capable of acting on a distal site. In *Lactobacillus rhamnosus* GR-1, the analogous protein to p75 is called major secreted protein-1 (MSP-1) [7]. Inactivation of MSP-1 in mutant strains causes a visible defect in cell separation, which is restored by plasmid complementation [7]. Using a cell-culture model for heart failure, phenylephrine-induced hypertrophic cardiomyocytes were co-cultured with several probiotic strains. We hypothesized that soluble factors produced by *L*. *rhamnosus* GR-1, including MSP-1, could attenuate the hypertrophic outcome of I/R injury and heart failure in cardiomyocytes.

## Materials and Methods

### Assess the effect of MSP-1 on hypertrophic cultured myocytes

The animal use protocol was approved by the Animal Use Subcommittee of the University Council on Animal Care from the University of Western Ontario. AUP #2013–031. Procedures adhered to the guidelines of the Canadian Council on Animal Care.

Based on preliminary co-culture experiments, we already know that hypertrophic myocytes grown in the presence of GR-1 and GR-1 supernatant have prolonged survival and improved morphology. Using an MSP-1 mutant strain of *L*. *rhamnosus* GR-1, CMPG10200 we determined the cell survival of hypertrophic myocytes in the presence and absence of MSP-1. To assess the effect of administration of additional probiotic strains on cultured cardiomyocytes, co-culture assays were developed using neonatal rat ventricular cardiomyocytes (NVCM). Primary NVCM cultures were isolated from 1–3 day old neonatal Sprague-Dawley rat pups sacrificed by decapitation (Charles River Canada, St Constant, Quebec, Canada) by lab staff of Dr. Morris Karmazyn as previously described [8]. For experiments, NVCM isolated from the same litter were pooled to represent one biological replicate (one “n” value).

NVCM were plated on Primaria (Corning, New York USA) culture dishes at optimal concentrations for each co-culture experiment: 3 × 10^4^ cells to allow room for growth and visualization for cell surface area measurements and 6 × 10^4^ cells to ensure sufficient RNA production for gene expression experiments. Two mL of warm culture medium was added to each dish and the cells were maintained at 37°C 5% CO_2_. After 48 hours, cells were washed with warm phosphate-buffered saline (PBS) and the culture media was changed daily thereafter. All reagents were tissue culture grade and filter sterilized or autoclaved. The NVCM cell culture medium was adjusted to a pH of 7.10. Cells were maintained for either 3 days for cell surface area experiments, or 5 days for gene expression experiments, before commencing each experiment.

### Probiotic cultures and conditions

Five probiotic strains were used for NVCM co-culture experiments: *Lactobacillus rhamnosus* GR-1, *Lactobacillus rhamnosus* CMPG10200, *Lactobacillus reuteri* RC-14, *Lactobacillus plantarum* 299v, and *Streptococcus salivarius* K12. *Lactobacillus rhamnosus* CMPG10200 is a mutant of *L*. *rhamnosus* GR-1 and was kindly provided by Dr. Sarah Lebeer, Department of Bioengineering, University of Antwerp, Belgium. The strain was constructed by insertational mutagenesis. Briefly, an internal fragment of the *msp1* gene was amplified by PCR and cloned into pCRII-TOPO vector (Invitrogen). The vector was digested with *Eco*RI and further ligated into an erythromycin-resistant vector. *E*. *coli* TOP10F was transformed using this ligation product and the suicide vector was then electroporated to *L*. *rhamnosus* GR-1. *L*. *rhamnosus* CMPG10200 was grown in MRS broth/agar with the addition of 5 μg/mL erythromycin. The Msp1 protein is a cell wall hydrolase that is necessary for daughter cell separation. Prior to receiving the strain, the absence of Msp1 knock-out was confirmed by Western blot using anti-Msp1 antiserum. Microscopic examination of the mutant also confirmed the absence of Msp1: While the mutant is able to grow to the same density as the wild-type (WT), there is a clear defect of cell wall separation visualized by bright-field microscopy.

The strains were resuscitated from -80°C freezer and 10 mL of the appropriate broth media was inoculated from a single isolated colony of each probiotic strain from an agar plate, and grown anaerobically in de Man, Rogosa and Sharpe (MRS; Becton Dickinson) media using the GasPak system (Becton Dickinson) at 37°C. The cultures were resuspended to 10^9^ CFU/mL,

To create the probiotic-conditioned media (PCM), 1 mL of the broth culture was placed in a clean, sterile, microcentrifuge tube and centrifuged at 12,000 *x g* at 4°C for 5 minutes. The supernatant was carefully collected without disturbing the bacterial pellet and transferred to a new, sterile microcentrifuge tube. This centrifugation step was repeated, and the supernatant was transferred to a new sterile microcentrifuge tube, to be used as the PCM treatment. Samples of the PCM treatments were plated onto MRS agar to ensure no bacterial cells could be detected by cultivation.

Aliquots of the PCM were also either heat denatured (HD) by incubation at 80°C for 30 minutes or incubated in a 1:1 trypsin-EDTA (Gibco, Burlington, Canada) solution at 37°C for 20 minutes. The trypsin-PCM (Tryp PCM) solution was then treated with a 0.05% trypsin soybean inhibitor *Glycine max* (Sigma Aldrich, Oakville, Canada) to eliminate trypsin activity on the NVCM culture. For filtered PCM treatments, the PCM was filtered using Centricon Plus-20 centrifugal filters according to manufacturer’s instructions (Millipore, Toronto, Canada). Both the filtrate and retentate were collected and applied as a treatment. The original bacterial pellet from the PCM treatment preparation was saved, washed with ice cold sterile PBS, and centrifuged at 12,000 *x g* at 4°C. The supernatant was discarded and the pellet was resuspended in 1 mL of sterile PBS. The pellet was then vortexed thoroughly and the suspension was used as the live probiotic cell treatment, with PCM removed. Aliquots of these cells were heat-killed (HK) by incubation at 80°C for 30 minutes, and plated onto MRS agar to ensure no live bacterial cells were present. All volumes of probiotic treatments used in the co-culture experiments were 50 μL, unless otherwise stated. The average concentration of all probiotic cultures used in each experiment was 10^9^ CFU/mL.

### Induction of hypertrophy and probiotic administration

To induce hypertrophy in NVCM, the α1-adrenergic receptor agonist phenylephrine (PE; Research Biochemicals Inc., Natick, MA) was applied to the cells. Following 3 days of culture for cell surface area experiments and 5 days for gene expression experiments, 10 μM PE was added to each dish. After gently swirling to distribute the PE, a probiotic treatment was immediately added to the NVCM in triplicate. The dishes were gently swirled again, and incubated at 37°C, 5% CO_2_ for 24 hours. The medium was removed from each dish and replaced with PBS. Aliquots of media containing probiotic cells from every experiment were spread-plated to determine viability.

### Cell surface area measurement and analysis

The physical effect of probiotic administration on hypertrophic NVCM was evaluated by taking cell surface area measurements. Cells were viewed and images were obtained using phase contrast microscopy with the Nikon Eclipse TE2000 inverted microscope (Nikon Instruments Inc., Mississauga, Canada). Surface area measurements of 50 random cells from at least 5 fields of view per dish (20x objective) were conducted blindly using the calibrated NIS-Elements BR software (Nikon Instruments Inc.). The average cell surface area per dish was used to calculate a total average cell surface area per treatment. The fold change in surface area was determined by dividing the treatment average by the control average. All experiments were repeated independently at least 5 times.

### Gene expression of hypertrophic markers in NVCM co-cultured with probiotics

In order to confirm that the change in cell surface area was due to an attenuation of hypertrophy, the gene expression of the hypertrophic markers ANP and α-skeletal actin (aSKA) were measured after a 24-hour treatment period. The analysis was performed using the real-time qPCR technique, and all experiments were repeated independently three times.

### RNA extraction and purification

Following the 24-hour treatment period, the medium was aspirated off each dish and stored in sterile a microcentrifuge tube at 4°C for later bacterial enumeration. 1 mL of TRIzol (Invitrogen, Burlington, Canada) was added to each dish. The cells were scraped thoroughly off each dish using a plastic cell scraper and the TRIzol mixture was transferred to an RNase-free 1.5 mL microcentrifuge tube, vortexed, and incubated at room temperature for 10 minutes. Two hundred μL of chloroform was added to each tube, vortexed vigorously, and incubated at room temperature for 10 minutes. The tubes were then centrifuged at 16,000 *x g* for 15 minutes at 4°C. 500 μL of the upper aqueous phase containing RNA was carefully transferred into a new RNase-free microcentrifuge tube. 500 μL of 100% ethanol was added to each tube containing the aqueous RNA and the tubes were vortexed for 20 seconds. 500 μL of the ethanol-RNA mixture was transferred to a PureLink RNA spin column (Life Technologies, Burlington, Canada). The column was centrifuged at 12,000 *x g* for 15 seconds, and the flow through was discarded. This was repeated until all of the ethanol-RNA mixture was passed through the column. Following the PureLink RNA mini kit protocol for binding, washing, and elution (Life Technologies), RNA was recovered and quantified using the NanoDrop 1000 spectrophotometer (Thermo Fisher Scientific, Burlington, Canada). Purity of RNA was quantified using 260/280 and 260/230 ratios. RNA quality cut-off was set at >1.75 and >1.8 for 260/280 and 260/230, respectively. RNA quality was also confirmed by viewing products via electrophoresis in a 1% agarose gel using 1x TAE stained with ethidium bromide, and viewed under UV light using AlphaImager (Alpha Innotech Corporation, San Jose, USA).

RNA samples falling under the quality limits underwent an ethanol precipitation protocol as follows: 3 μL of sodium acetate and 90 μL of 100% ethanol were added to each RNA sample for overnight precipitation at -20°C. The next day, RNA samples were centrifuged at 16,000 *x g* for 20 minutes at 4°C. The supernatant was discarded and the pellet containing crude RNA was resuspended in ice-cold 70% nuclease-free ethanol, vortexed, and centrifuged at 16,000 *x g* for 20 minutes at 4°C. This step was repeated once more. All the ethanol was removed from each tube using a fine pipette tip, and the tubes were left open for 1 minute to evaporate the last of the ethanol. The crude RNA was then resuspended in 15 μL nuclease-free water, and quality was assessed using the NanoDrop 1000 spectrophotometer, as described above. RNA samples were stored at—20°C until further processing.

### Reverse transcription

Samples of RNA ranging from 300 ng– 2 μg were used as a template for RT PCR. cDNA was synthesized using the High Capacity cDNA Reverse Transcription Kit (Applied Biosystems, Burlington, Canada). Ten μL of the RT master mix was combined and mixed with 10 uL of RNA sample (or 10 uL of nuclease-free water for the negative control) in an RNAase-free 96-well plate, creating a 20 μL reaction. The PCR reaction was carried out in an Eppendorf Mastercycler PCR machine (Eppendorf, Mississauga, Canada). cDNA concentration was measured on all negative controls and 3 random cDNA samples using a Qubit 2.0 fluorometer (Life Technologies) to confirm successful RT. The cDNA was then diluted into 340 μL of nuclease-free water and stored at -20°C until further use.

### Quantitative real-time PCR

qPCR was used to evaluate the gene expression of the hypertrophic markers atrial natriuretic peptide (ANP) and α-skeletal actin (aSKA), relative to a housekeeping gene, in this case 18S rRNA. ANP, aSKA and 18S rRNA primer sequences were designed using the Primer-BLAST tool from the National Center for Biotechnology Information (NCBI) [9]. Gene Primer Sequence for aSKA (NM_019212.2):

Forward: 5’-CAGAGTCAGAGCAGCAGAAACT-3’, Reverse: 5’-GTTGTCACACACAAGAGCGG-3’, Product size: 71 base pairs (BP).

Sequence for ANP (NM_012612.2):

Forward: 5’-CCCTCCGATAGATCTGCCCT-3’, Reverse: 5’-TTCGGTACCGGAAGCTGTTG-3’, Product size: 148 BP. Sequence for 18S rRNA (NR_046237.1): Forward: 5’-GTAACCCGTTGAACCCCATT-3’, Reverse: 5’-CCATCCAATCGGTAGTAGCG-3’, Product size: 148 BP. The primers were stored in a stock solution containing 800 nM of both forward and reverse primer. Prior to running the cDNA samples, the primers were validated using serial dilutions of a positive control cDNA sample. Five μL of each serial dilution was combined in triplicate with 10 μL Power SYBR Green Master Mix (Life Technologies), and 5 μL of the 800 nM primer stock solution. The PCR reaction was then carried out in a 384-well reaction plate using the 7900 HT Sequence Detection System (SDS) and primer efficiencies were determined using SDS 2.3 Sequencing Software (Applied Biosystems, Life Technologies). The efficiencies for 18S rRNA, ANP and aSKA respectively were 97.24, 97.50 and 98.03%. The PCR products were then viewed via electrophoresis in a 3% agarose gel using 1x TAE stained with ethidium bromide, and viewed under UV light using AlphaImager (Alpha Innotech Corporation).

After primer validation, cDNA samples were used as template for qPCR reactions. 5 μL of each cDNA sample was combined in triplicate with 10 μL Power SYBR Green Master Mix (Life Technologies), and 5 μL of 800 nM primer stock solution. Each reaction was carried out in triplicate for all 3 primers in a 384-well reaction plate using the same machine and method as above.

### Real-time quantitative PCR data analysis

Analysis was carried out using RQ Manager 1.2 Data Analyzer (Applied Biosystems, Life Technologies). Relative gene expression of aSKA and ANP was determined using 18S rRNA as the endogenous control. Gene expression in a treatment cDNA sample measured in terms of relative quantification (RQ), using the comparative threshold cycle (Ct) method. The Ct of the PCR reaction in which aSKA or ANP was detected was compared to the Ct of detection for 18S rRNA. The total difference in Ct over 40 cycles of PCR is then used to determine the RQ of a sample. RQ represents the fold change in gene expression compared to a calibrator (untreated control cDNA sample). The RQ of the calibrator = 1. The standard deviation (SD) of Ct values was used to determine the quality of the technical replicates in each sample. If the SD was over 0.25, the RQ value for that sample was considered unreliable and was not used in the data analysis.

### Statistical Analysis

All statistical analyses were performed using GraphPad Prism 5 and statistical analysis reports for all NVCM experiments are provided in the appendix. All data were analyzed using a 1-way ANOVA followed by a post hoc Tukey test. Differences were considered significant when *P* < 0.05. Statistical analysis for the next-generation sequencing data was performed using Uclust^187^, QIIME^190^, UniFrac^191^, and a modified version of a data analysis pipeline [10,11].

For NVCM experiments, technical triplicates for each experiment were performed on at least 3 biological replicates. Data reported are means of biological replicates ± standard deviation. Differences between means of the treatments were compared using a 1-way ANOVA followed by a post hoc Tukey test, or by a Student’s *t-*test. Differences were considered significant when *P* < 0.05.

## Results

### Effect of phenylephrine administration on hypertrophy in NVCM

In order to determine the hypertrophic response to PE administration, and to rule out that MRS broth affects the growth of cultured NVCM, 50 μL of sterile MRS broth was added NVCM culture with or without PE. Twenty-four hour PE exposure induced an average 1.41-fold increase in NVCM surface area, compared to untreated control cells. PE with MRS broth induced a 1.35-fold increase in cell surface area. The difference in cell surface area compared to both of these treatments was statistically significant (*P* < 0.05), but difference between the two PE treatments without MRS broth was not significant (*P* = 0.52). Without PE, the average fold change in surface area of cells cultured with MRS broth compared to untreated cells after 24 hours was 1.02. A student’s *t*-test, was performed to determine the difference between MRS broth-treated and untreated cells. The difference was not significant (*P* = 0.75).

The gene expression of the hypertrophic markers ANP and aSKA was evaluated to confirm a hypertrophic response in NVCM exposed to PE. The administration of PE induced a 1.91-fold and 1.51-fold increase in gene expression of ANP and aSKA, respectively. The administration of PE with 50 μL of sterile MRS broth induced a 2.01-fold increase in gene expression of ANP, and a 2.10-fold of aSKA. The difference in ANP expression with both PE and PE + MRS broth compared to untreated cells was statistically significant (*P* < 0.05), but the difference between the two PE treatments was not statistically significant. There was no statistically significant difference in expression of aSKA between PE and untreated cells (*P* = 0.0921), likely because of the high standard deviation in the PE group. There was, however, a significant difference in aSKA expression between MRS broth and PE + MRS broth (*P* < 0.01).

The administration of MRS broth without PE produced a 0.96 and 0.79-fold change in gene expression of ANP and aSKA, respectively, over 24 hours, compared to untreated cells. A student’s *t*-test was performed to determine the difference in gene expression between untreated and MRS broth-treated NVCM. The expression of ANP and aSKA were both found to be not significantly different (*P* = 0.1160 and *P* = 0.1909, respectively). These results confirm that PE administration induced hypertrophy over 24 hours in NVCM. The presence of sterile MRS broth has no effect on the growth of NVCM.

**Fig 1:**
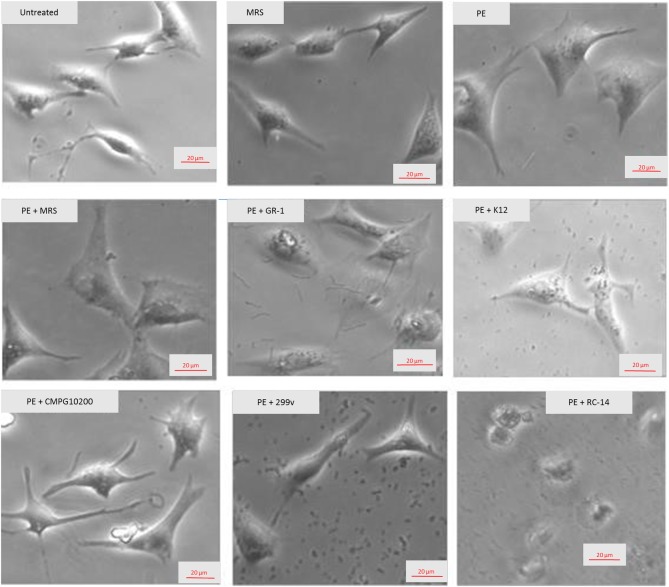
Representative micrographs illustrating NVCM exposed to PE alone or with probiotics. The control NVCM were either untreated, or co-cultured with MRS broth. Original magnification = 20×.

NVCM co-cultured with *L*. *rhamnosus* GR-1, *L*. *plantarum* 299v, and *S*. *salivarius* K12 had normal cell morphology after 24 hours, as indicated by the representative micrographs (Fig 1). In addition, the cells were spontaneously beating, an indication (however not a prerequisite) of NVCM viability. The probiotics were also successfully isolated from the culture media 24 hours after co-culture with NVCM, indicating that they were not susceptible to the penicillin/streptomycin antibiotic solution in the NVCM culture medium. The majority of NVCM co-cultured with *L*. *reuteri* RC-14 did not survive the 24-hour treatment period, as evidenced by loss of cell attachment and abnormal morphology. A scarce number of NVCM cultured with *L*. *reuteri* RC-14 were beating after the 24-hour treatment period. The *L*. *reuteri* RC-14 cells also did not survive the treatment period, as they were unsuccessfully isolated from the culture medium.

### *L*. *rhamnosus* GR-1 administration inhibited PE-induced hypertrophy in NVCM

**Fig 2:**
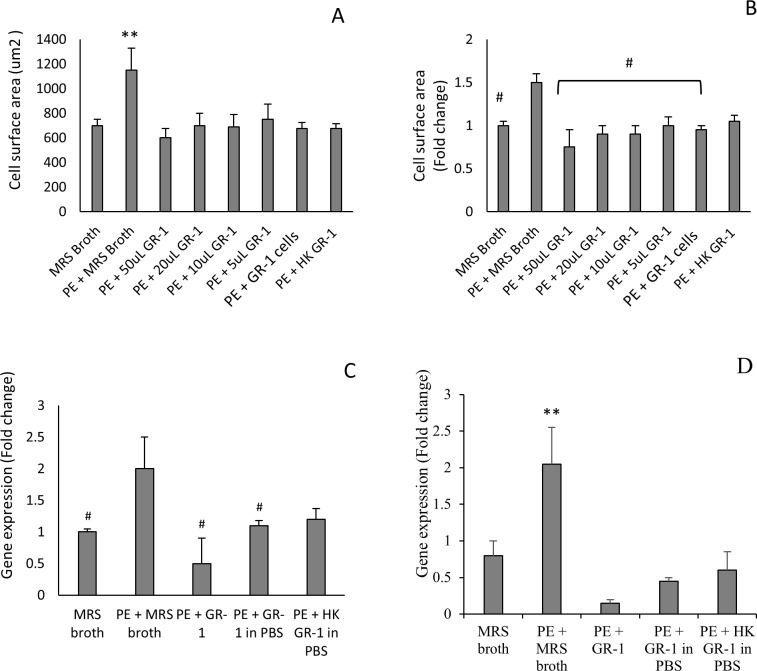
*L*. *rhamnosus* GR-1 inhibits PE-induced hypertrophy in NVCM. **A**: The NVCM surface area following 24 hour co-culture with GR-1 (n = 5). PE + GR-1 = GR-1 bacteria in PBS; HK GR-1 = GR-1 bacteria heat killed. **B**: The fold change in NVCM surface area compared to untreated (control) cells, following 24 hour co-culture with GR-1 (n = 5). **C, D**: The fold change in gene expression of ANP (C) and aSKA (D) in NVCM (n = 3) respectively. Unless otherwise stated, all volumes of GR-1 treatments were 50 μL (1 x 10^9^ CFU/mL stock culture). *P < 0.05 and **P < 0.01 compared to all other treatments. *P < 0.05 compared to PE + MRS broth. Error bars indicate the standard deviation.

When *L*. *rhamnosus* GR-1 was co-cultured with NVCM immediately following PE administration, there was no increase in NVCM surface area compared to untreated cells. The inhibition of hypertrophy was confirmed by the gene expression of ANP and aSKA relative to untreated cells. The expression of both hypertrophic markers was significantly reduced when NVCM cells exposed to PE were co-cultured with *L*. *rhamnosus* GR-1 (*P*< 0.05). To confirm the presumption that live probiotic cells are required to confer an anti-hypertrophic effect, *L*. *rhamnosus* GR-1 cells were HK by incubation at 80°C for 30 minutes. HK GR-1 cells and live GR-1 cells had the same average cell surface area, however, when the data was normalized by calculating the fold change in cell surface area, there was a loss of anti-hypertrophic activity in HK GR-1 cells. The difference in gene expression of ANP in PE-treated NVCM without HK GR-1 cells and PE-treated NVCM with HK GR-1 cells was not statistically significant. For both ANP and aSKA, there was a slight increase in gene expression with HK GR-1 cells, compared to live *L*. *rhamnosus* GR-1. The increase, however, was not statistically significant. (*P* = 0.35 and 0.36 for ANP and aSKA respectively). These data are summarized in Fig 2.

**Fig 3:**
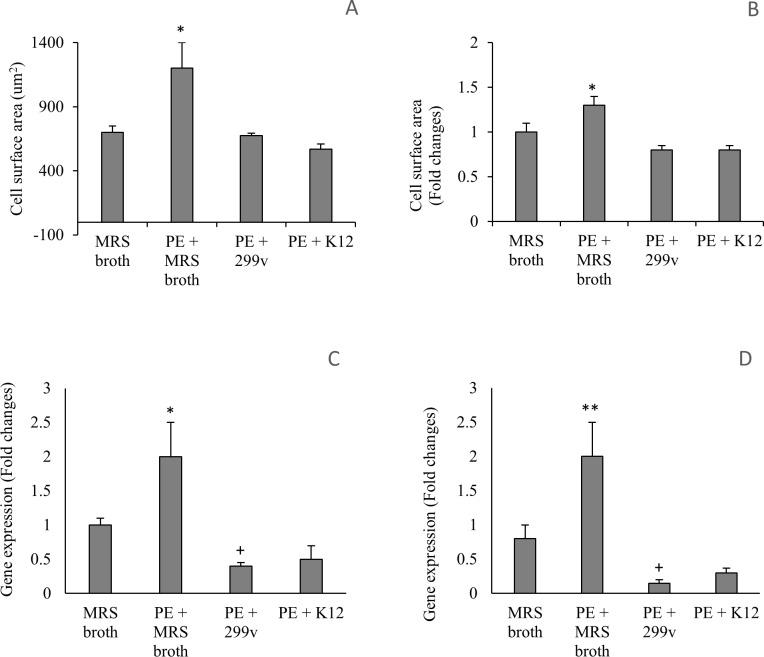
*L*. *plantarum* 299v and *S salivarius* K12 inhibits PE-induced hypertrophy in NVCM. **A**: The NVCM surface area following 24 hour co-culture with 299v and K12 (n = 5). **B**: The fold change in NVCM surface area compared to untreated cells, following 24 hour co-culture with 299v and K12 (n = 5). **C, D**: The fold change in gene expression of ANP (C) and aSKA (D) compared to untreated NVCM after 24 hour co-culture with 299v and K12 (n = 3). All volumes of 299v and K12 treatments were 50 μL (1 x 10^9^ CFU/mL stock culture). *P < 0.05 and **P < 0.01 compared to all other treatments. ^+^*P* < 0.05 compared to MRS broth. Error bars indicate the standard deviation.

Based on the cell surface area and gene expression of ANP and aSKA after 24-hour co-culture, *L*. *plantarum* 299v and *S*. *salivarius* K12 also inhibited the PE-induced hypertrophy in NVCM. There was a significant decrease in ANP and aSKA gene expression in NVCM co-cultured with *L*. *plantarum* 299v compared to the control (*P* < 0.05) (Fig 3).

### MSP-1 is not required to inhibit PE-induced increase in NVCM surface area

**Fig 4:**
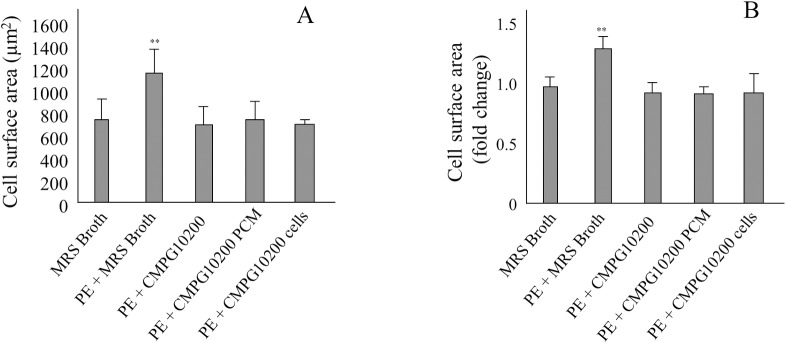
*L*. *rhamnosus* GR-1 MSP-1 knock out strain CMPG10200 inhibits PE-induced hypertrophy in NVCM. **A.** The NVCM surface area following 24 hour co-culture with CMPG10200 (n = 5). **B.** The fold change in NVCM surface area compared to untreated cells, following 24 hour co-culture with CMPG10200 (n = 5). All volumes of CMPG treatments were 50 μL (1 x 10^9^ CFU/mL stock culture). ^**^P<0.01 compared to all other treatments. Error bars indicate the standard deviation.

MSP-1 produced by *Lactobacillus rhamnosus* was of interest because it has shown to protect against ischemic injury and stress induced apoptosis in the heart and small intestine. We tested a mutant MSP-1 knock out *L*. *rhamnosus* GR-1 strain, CMPG10200, but there was a similar inhibition of PE-induced increase in NVCM surface area as for the WT *L*. *rhamnosus* GR-1 strain (Fig 4). This suggests that the protein MSP-1 is not required for preventing cell surface area increase in NVCM exposed to PE, and therefore, gene expression analysis using this strain was not pursued.

### PE-induced hypertrophy in NVCM is attenuated by PCM treatment alone

**Fig 5:**
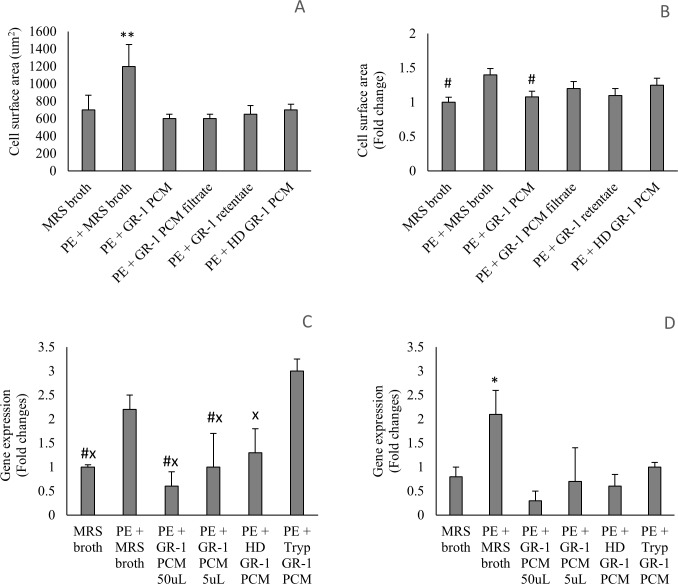
PCM inhibits the PE-induced hypertrophy in NVCM. **A**. NVCM surface area following 24 hour co-culture with various PCM treatments (n = 5). **B**: The fold change in NVCM surface area compared to untreated cells, following 24 hour co-culture with various PCM treatments (n = 5). **C, D**: The fold change in gene expression of ANP (C) and aSKA (D) compared to untreated NVCM after 24 hour co-culture with various PCM treatments (n = 3). Unless otherwise stated, all PCM volumes were 50 μL. *P < 0.05 and **P < 0.01 compared to all other treatments^. #^P < 0.05 compared to PE + MRS broth. ^×^P < 0.05 compared to PE + Tryp GR-1 PCM. Error bars indicate the standard deviation. HD denotes heat denatured.

When NVCM were treated with GR-1 PCM void of any live bacteria, the PE-induced increase in cell surface area and gene expression of ANP and aSKA was significantly attenuated, similar to treatments with live probiotic cells (*P* < 0.05). Attempts were made to elucidate the size of the potential anti-hypertrophic factor by centrifugally filtering the PCM. While the overall surface area of cells indicates that both the filtrate and retentate retained an anti-hypertrophic effect, the fold-change in cell surface area indicates that this effect was lost. We then heat denatured the PCM by a 30 minute incubation at 80°C and trypsin treatment, resulting in a loss of anti-hypertrophic activity of the PCM, as indicated by the fold change in NVCM surface area and ANP gene expression. In NVCM co-cultured trypsin-treated PCM, the attenuation of PE-induced increase in ANP gene expression was lost. There was an increase in PE-induced aSKA gene expression with HD and trypsin-treated PCM compared to regular PCM, however the difference was not significant. Heat denatured PCM significantly attenuated the PE-induced increase in aSKA expression (*P* < 0.05). These data are summarized in Fig 5.

## Discussion

This study showed that probiotic *L*. *rhamnosus* GR-1 and its supernatant can attenuate hypertrophy of cardiomyocytes. This supports the previous animal study showing that administration of the GR-1 probiotic strain improved recovery from ischemic injury [1]. Cell lines that are physiologically accustomed to bacterial exposure, such as gut epithelial cells, gingival cells, skin cells, or vaginal cells, may be tolerant to bacterial co-culture *in vitro*. However, NVCM cells are not conventionally exposed to bacteria and antibiotics are added to the culture media. The addition of 5 x 10^7^ CFU of *L*. *rhamnosus* GR-1, *L*. *plantarum* 299v, and *S*. *salivarius* K12 to NVCM had no adverse effects on cell viability or function. In fact, with the daily replacement of cell culture media and *L*. *rhamnosus* GR-1 cells, spontaneously beating NVCM were maintained for as long as seven days, which is the recommended duration of culture for these primary cells [12, 13]. The antibiotics did not affect *L*. *rhamnosus* GR-1 cell viability.

In efforts to elucidate the mechanism for the attenuation of heart failure by GR-1 seen in our previous animal studies [1], PE was used to induce hypertrophy in NVCM. This α-adrenergic receptor agonist is an agent commonly used in models of cardiac hypertrophy relevant to heart failure [14–16]. The effect of PE on NVCM was evaluated by calculating the cell surface area and gene expression of the hypertrophic markers ANP and aSKA 24 hours after PE exposure. To closely mimic the design of the coronary artery ligation animal studies we previously performed [1], each probiotic treatment was administered to NVCM immediately following PE exposure. Physical analysis on the change in cell surface area indicates that *L*. *rhamnosus* GR-1, *L*. *plantarum* 299v, and *S*. *salivarius* K12 administration blocks PE-induced hypertrophy in NVCM over 24 hours, and normal NVCM morphology and function is maintained. This phenotype was seen for a range of concentrations of *L*. *rhamnosus* GR-1, suggesting a strong potency of the treatment. The anti-hypertrophic activity of *L*. *rhamnosus* GR-1 was confirmed by a significant attenuation of the PE-increased gene expression of both ANP and aSKA. This supported the hypothesis that the cardiac benefits conferred by probiotics *in vivo* and *in vitro* is not strain specific. Interestingly, NVCM co-cultured with *Lactobacillus reuteri* RC-14, a vaginal isolate, generally did not survive the assays.

It was presumed that live probiotic cells are required to confer salutary cardiac benefits. Dead *L*. *rhamnosus* GR-1 or *L*. *plantarum* 299v cells were not administered in the previous coronary artery ligation studies, however in NVCM co-culture experiments, HK *L*. *rhamnosus* GR-1 cells were administered, and a loss of anti-hypertrophic activity was noted, as determined by the fold change in cell surface area and ANP gene expression. However, the difference in the NVCM hypertrophy of live GR-1 cells compared to HK GR-1 cells was not statistically significant for all parameters measured. The possibility that *L*. *rhamnosus* GR-1 formed a heat-stable biofilm and thus undetectable live organisms were still present, is unlikely as the GR-1 strain is a poor biofilm former and no lactobacilli survived the heat treatment. Another possibility is that anti-hypertrophic factors produced by *L*. *rhamnosus* GR-1 were present despite the heat treatment. Further experiments are warranted to explain the results.

While cardiomyocytes will not encounter such high densities of live probiotic cells *in vivo*, soluble factors produced by the lactobacilli may cross the gut epithelial barrier into the blood and reach the heart. There is evidence of a cytoprotective effect of p75 in intestinal and hepatic cells and evidence that *L*. *rhamnosus* produces a soluble protein with anti-apoptotic activity that is directly protective against I/R-associated injury to the heart [5]. But, using a knock out mutant strain for this protein, MSP-1, in *L*. *rhamnosus* GR-1 CMPG10200, we found no effect on the NVCM surface area. Thus, we do not believe the therapeutic effect of *L*. *rhamnosus* GR-1 in our animal and cell models for heart failure and cardiac hypertrophy to be mediated by MSP-1.

When lactobacilli whole cells were completely removed, the PCM conferred a similar effect against PE-induced hypertrophy in terms of cell surface area and gene expression of ANP and aSKA, albeit to a lesser magnitude than the whole cell preparation. This indicated that *L*. *rhamnosus* GR-1 produces a factor that prevented PE-induced hypertrophy in NVCM. It was hypothesized that the responsible factor was a protein, and treated the PCM with trypsin, with the result that the effects on surface area and ANP gene expression were completely lost. While this effect was not as marked in aSKA, there was a slight increase in gene expression compared to regular PCM. If a molecular marker of hypertrophy in cardiomyocytes had been available, it would have been interesting to use it to measure impact, rather than solely relying on surface area measurements.

The role of proteins derived from *Lactobacillus rhamnosus* GR-1in preventing hypertrophy was further examined by centrifugal filtration based on molecular size exclusion. Filtration of the PCM through 20 nm pore size filter did not result in a significant difference in the average NVCM surface area compared to untreated cells, however the fold change in surface area indicated that anti-hypertrophic activity was lost in both the filtrate and retentate. This suggested that there is may be more than one protein responsible for attenuating hypertrophy which cannot be effectively separated by size. It was hoped to elucidate the heat sensitivity of these potential proteins by incubating the PCM at a high temperature, typically heat stable proteins are smaller in mass. Trypsin acts indiscriminately on proteins by cleaving peptide chains, but heat denaturing a solution may result in variable outcomes, depending on the nature of the proteins present. Large proteins are typically heat sensitive and denature at lower temperatures than smaller ones. Small, heat stable proteins (less than 20 kDa) have the potential to maintain function after heat incubation and are reported to be produced by various lactobacilli strains [17,18]. In these experiments, heat denatured PCM did not significantly attenuate the PE-induced increase in cell surface area (fold change) or increase in ANP gene expression, indicating a loss in anti-hypertrophic activity. This may be due to the destabilization of the protein(s) by the heat treatment. There was no difference in gene expression of aSKA with heat denatured PCM compared to regular PCM. These mixed results might suggest that the PCM affects ANP expression differently than aSKA, or that is more than one protein involved.

There was a difference in the magnitude of the change of gene expression in ANP compared to aSKA, depending on the nature of the PCM treatment. Both of these hypertrophic markers are expressed during prenatal cardiac development and then are downregulated after birth [19,20]. Baseline expression of ANP and aSKA in NVCM is higher than adult cardiomyocytes and gene expression can be induced in both cell types by hormonal and hemodynamic stimuli as well as pharmacological hypertrophic agents [19]. The signaling mechanisms and pathways for ANP and aSKA differ from one another, and they are not equally as responsive to some stimuli. As discussed earlier, ANP is a vasodilator that becomes active in response to hypertension [19] and aSKA is involved in muscle formation and is associated with cellular growth [20]. Hypertrophic phenotypes, therefore, may feature activated ANP expression, while aSKA is not induced [20]. The difference lies in the nature of the stimulus used to induce hypertrophy.

While PE has shown to induce both ANP and aSKA expression in several models of cardiac hypertrophy [15, 20, 21], other factors may affect the complex and diverse signal transduction pathways. In the present studies, it is possible that PCM interferes with the signaling pathway for PE-induced aSKA gene expression, while ANP is unaffected.

In summary, this study provides further evidence that probiotic lactobacilli have the potential to positively influence cardiac function.
